# Intra-Tumor Heterogeneity Revealed by Mass Spectrometry Imaging Is Associated with the Prognosis of Breast Cancer

**DOI:** 10.3390/cancers13174349

**Published:** 2021-08-27

**Authors:** Marta Gawin, Agata Kurczyk, Joanna Niemiec, Agata Stanek-Widera, Aleksandra Grela-Wojewoda, Agnieszka Adamczyk, Magdalena Biskup-Frużyńska, Joanna Polańska, Piotr Widłak

**Affiliations:** 1 Maria Skłodowska-Curie National Research Institute of Oncology, Gliwice Branch, 44-102 Gliwice, Poland; marta.gawin@io.gliwice.pl (M.G.); agata.kurczyk@io.gliwice.pl (A.K.); aswidera502@gmail.com (A.S.-W.); magdalena.biskup-fruzynska@io.gliwice.pl (M.B.-F.); 2 Maria Skłodowska-Curie National Research Institute of Oncology, Kraków Branch, 31-115 Kraków, Poland; joanna@eikon.pl (J.N.); agw10@interia.pl (A.G.-W.); agnieszka.adamczyk@onkologia.krakow.pl (A.A.); 3 Medical College of Rzeszow University, 35-959 Rzeszów, Poland; 4 Faculty of Medicine, University of Technology in Katowice, 40-555 Katowice, Poland; 5 Silesian University of Technology, 44-100 Gliwice, Poland; joanna.polanska@polsl.pl

**Keywords:** HER2-positive breast cancer, intra-tumor heterogeneity, mass spectrometry imaging, prognosis, long-term outcome, tumor microenvironment

## Abstract

**Simple Summary:**

The coexistence of genetically distinct cancer cell clones, their phenotypic plasticity, and the presence of different constituents of the tumor microenvironment create intra-tumor heterogeneity, which affects cancer development and its response to therapy. We observed that a higher degree of phenotypic heterogeneity revealed by mass spectrometry imaging was associated with a favorable outcome in HER2-positive breast cancer. This phenomenon putatively reflects the presence of heterotypic components of the microenvironment, which could facilitate the response to anticancer treatment.

**Abstract:**

Intra-tumor heterogeneity (ITH) results from the coexistence of genetically distinct cancer cell (sub)populations, their phenotypic plasticity, and the presence of heterotypic components of the tumor microenvironment (TME). Here we addressed the potential association between phenotypic ITH revealed by mass spectrometry imaging (MSI) and the prognosis of breast cancer. Tissue specimens resected from 59 patients treated radically due to the locally advanced HER2-positive invasive ductal carcinoma were included in the study. After the on-tissue trypsin digestion of cellular proteins, peptide maps of all cancer regions (about 380,000 spectra in total) were segmented by an unsupervised approach to reveal their intrinsic heterogeneity. A high degree of similarity between spectra was observed, which indicated the relative homogeneity of cancer regions. However, when the number and diversity of the detected clusters of spectra were analyzed, differences between patient groups were observed. It is noteworthy that a higher degree of heterogeneity was found in tumors from patients who remained disease-free during a 5-year follow-up (*n* = 38) compared to tumors from patients with progressive disease (distant metastases detected during the follow-up, *n* = 21). Interestingly, such differences were not observed between patients with a different status of regional lymph nodes, cancer grade, or expression of estrogen receptor at the time of the primary treatment. Subsequently, spectral components with different abundance in cancer regions were detected in patients with different outcomes, and their hypothetical identity was established by assignment to measured masses of tryptic peptides identified in corresponding tissue lysates. Such differentiating components were associated with proteins involved in immune regulation and hemostasis. Further, a positive correlation between the level of tumor-infiltrating lymphocytes and heterogeneity revealed by MSI was observed. We postulate that a higher heterogeneity of tumors with a better prognosis could reflect the presence of heterotypic components including infiltrating immune cells, that facilitated the response to treatment.

## 1. Introduction

Genetic alterations gained during cancer development are usually (sub)clonal events, and therefore solid cancers evolve to mosaic entities composed of a mixture of cells with different genomes. The resulting repertoire of genetically distinct cancer cell populations is referred to as intra-tumor heterogeneity (ITH) [1,2,3]. Genetic and epigenetic ITH could hypothetically be reflected in all distinguishable phenotypic features, such as cellular morphology, gene and protein expression, metabolism, as well as angiogenic, proliferative, immunogenic, and metastatic potential. Furthermore, a substantial component of tumor heterogeneity reflects the differentiation of cancer stem cells (CSC), epidermal to mesenchymal transition (EMT), and the phenotypic plasticity induced by interactions between cancer cells and different local microenvironments. Moreover, in addition to the “core” ITH characteristics of actual cancer cells (or rather sub-clones of cancer cells), tumor heterogeneity is further increased by the presence of heterotypic elements of its microenvironment (TME), including immune cells, connective tissues, microvasculature, etc. [3,4,5]. ITH facilitates the natural evolution of cancer that drives the process of tumor progression. Further, ITH could affect the effectiveness of anti-cancer treatment due to the selection of the resistant sub-clones initially present in a tumor, or due to the induction of new resistant sub-clones upon treatment. Despite the fundamental relevance of ITH for cancer progression and response to treatment, its clinical implications remain loosely defined. Although it has been hypothesized that ITH would be associated with poorer clinical outcomes in cancer patients, supportive evidence has long been limited to specific contexts of resistance to the therapy of metastatic cancers [6,7,8,9]. Nevertheless, it is not clear whether ITH might be a universal and independent determinant of patients’ outcome. A few studies used the pan-cancer TCGA dataset to show an association between the survival outcome in multiple cancer types and the degree of genetic ITH that was modeled based on the integration of the SNP array copy number and SNP mutation data from the whole-exome sequencing in large cohorts of patients. These reports showed that a high level of genetic ITH was generally associated with poorer survival across diverse types of cancers [10,11]. 

Despite the fundamental importance of ITH, surprisingly few experimental data were collected with direct relevance to this phenomenon. The fact that ITH has been long under-researched is related to serious limitations of the analytical approaches that could be implemented in this field of research. Currently, two “omics” approaches could potentially be used to study this phenomenon in the context of the actual histology of solid cancers. One of them is spatially resolved transcriptomics, that includes different methods based on in situ/in-tissue single-cell sequencing [12,13]. This approach, although powerful and dynamically developing (nominated the “Method of the Year 2020” [14]) is limited to nucleic acids. On the other hand, mass spectrometry imaging (MSI), which combines the analytical potential of mass spectrometry with the ability to scan series of pixels across the surface of tissues, enables the targeting of a wide range of molecules (proteins, lipids, metabolites, drugs) [15,16]. The mass profiles revealed by MSI can be spatially resolved and annotated with morphological and histological structures. Therefore, MSI can be used in studies aimed at the contribution of heterotypic material in solid tumors [17,18,19,20]. However, although MSI has been widely used in cancer research within the last decade, only a few papers refer to its utilization in the study of the phenomenon of ITH. Most of them were rather methodological and were focused on the development and optimization of tools used for data analysis [21,22,23,24]. In a few papers, MSI-guided microproteomics was employed to identify proteins characteristic of heterogeneous areas of breast cancers [25,26]. Moreover, an association between the heterogeneity of breast tumors and the presence of concomitant lymph node metastases was reported using this approach [24]. However, MSI has not been employed to analyze ITH in the context of the actual prognosis of breast cancer. Here we analyzed a set of HER2-positive breast tumors collected during the primary surgery, aimed at looking for a hypothetical association between phenotypic ITH and a long-term outcome. The unsupervised segmentation of MSI images was implemented to reveal the molecular heterogeneity of cancer regions and hypothetical differences between groups of patients where no evidence of disease or progressive disease was observed during a five-year follow-up.

## 2. Materials and Methods

### 2.1. Clinical Material

Postoperative tissue collected during a mastectomy or a breast-conserving surgery and stored as formalin-fixed paraffin-embedded material was used (the surgery was performed in the years 2007–2012). The tissue material was re-inspected and verified by experienced pathologists before the study. Samples of 59 females (aged from 40 to 78 years, median 57) with locally advanced HER2-positive invasive ductal carcinoma were included. Tumors with sizes between 1 cm and 5 cm (T1c and T2) were selected. Twenty-seven patients had no metastases in regional lymph nodes (N0), while in 32 patients cancer cells were detected in regional lymph nodes collected during lymphadenectomy (N1-N3). The majority of patients received adjuvant doxorubicin/cyclophosphamide/taxane-based chemotherapy supplemented with trastuzumab (hormonotherapy was applied if tumors had the expression of estrogen or progesterone receptors). No evidence of diseases was observed in 38 patients during at least a 5-year follow-up (ND group). Distant metastases were diagnosed in 21 patients during the same follow-up (progressive disease; PD group); the median time to progression was 35 months (major metastatic sites: lung—8 pts. and bones—5 pts.). Table 1 presents more detailed information about the compared groups.

### 2.2. Tissue Preparation for MALDI MSI

FFPE blocks were cut into 5 µm sections with the use of a rotary microtome (Microm HM 304E, Microm International GmbH, Walldorf, Germany) and placed onto ITO glass slides (Bruker Daltonik, Bremen, Germany) coated with poly-L-lysine; additionally, a subsequent serial section was placed on a regular glass slide and further stained with hematoxylin and eosin for histopathological evaluation; this serial section was used for the delineation of the cancer region of interest (ROI). Cancer ROI was marked on a section used for an MSI measurement after image co-registration (an example of a stained section with a delineated cancer ROI is illustrated in Supplementary Figure S1). The sections on ITO slides were left to dry at 37 °C overnight and then at 60 °C for 1 h, and subjected to paraffin removal by consecutive washing in a series of solvents, as reported in Supplementary Protocol 1. Then, the dewaxed sections were subjected to heat-induced antigen retrieval with the use of the Decloaking Chamber NxGen (Biocare Medical, Pacheco, CA, USA) according to Supplementary Protocol 2. This step was followed by on-tissue trypsin digestion. Sequencing grade modified trypsin (Promega, Madison, WI, USA) was deposited onto a glass slide using a SunCollect device (SunChrom GmbH, Friedrichsdorf, Germany) operated in the spraying mode, according to Supplementary Protocol 3. The slide was incubated for 18 h at 37 °C in a humid chamber filled with water, then dried in a vacuum desiccator for 30 min. Next, a solution of α-cyano-4-hydroxycinnamic acid (HCCA) was sprayed onto the slide with the use of the SunCollect device according to Supplementary Protocol 3.

### 2.3. MALDI MSI Measurements

MSI measurements were performed with the use of an ultrafleXtreme MALDI-ToF mass spectrometer (Bruker Daltonik, Bremen, Germany) according to a previously described method [27]. Mass spectra were acquired in the positive reflectron mode within an *m/z* range of 600–3500; 400 shots were collected from each ablation position with random walk activated (40 shots at raster spot). The raster width was set at 100 µm. The tissue sections were measured individually in random order.

### 2.4. Spectra Processing

The acquired MSI spectra were preprocessed by applying a series of computational steps that included mass channels unification, baseline subtraction, outlying spectra identification, peak alignment, and TIC normalization as described elsewhere [28]. The spectral components were detected using the Gaussian mixture modeling as described in detail in [29]. The resulting MSI dataset consisted of 377,310 spectra extracted from all cancer ROIs (59 imaged tissue specimens), each featuring 2527 components. The detailed procedure of spectra processing is provided in Supplementary Protocol 4. 

### 2.5. Statistical Analyses

A pairwise similarity index was calculated to assess the similarities between spectra from each cancer ROI [30]. Computed similarity values were plotted as cumulative distribution functions to visualize the differences of intra-ROI heterogeneity with relevance to patients’ groups. The deglomerative divisive iK-means (DivIK) algorithm with region-driven feature selection was used for unsupervised molecular image segmentation [23,31]. Spectra from all 59 cancer ROIs were analyzed together. The detailed procedure of image segmentation is provided in Supplementary Protocol 5. Cohen’s [d] effect size analysis was applied to indicate discriminatory components between pairwise compared sets of spectra (the [d] value was defined as the difference between the mean abundance of a component divided by the pooled standard deviation for the two compared groups of spectra [32]). Simpson’s diversity index [33] defined the probability of two randomly selected spectra from one ROI belonging to different clusters. Due to violations of the assumptions for the applicability of parametric tests, the nonparametric Wilcoxon rank-sum test, or the Kruskal–Wallis test were applied to determine differences in the number of clusters, Simpson’s diversity index, and cluster sizes if two or four groups were compared, respectively. The Kruskal–Wallis test was followed by the posthoc Conover test for pairwise comparisons [34]. Moreover, the eta-squared effect size was calculated for Kruskal–Wallis, and Pallant’s [r] effect size [35] for the Wilcoxon and Conover tests. 

### 2.6. Protein Identification by LC-MS/MS 

The identification of the proteins present in the lysates obtained from the analyzed tissues was performed with the use of the ultrafleXtreme mass spectrometer and a Proxeon EASY-nLC nano-liquid chromatograph coupled with a PROTEINEER fcII fraction collector (Bruker) as described in detail elsewhere [36]. The material included in this analysis consisted of consecutive FFPE tissue sections (5 × 10 µm) from five randomly selected patients; the selected tissue contained ca. 50% of cancer cells. The detailed protocol for the preparation of the tissue lysates is given in the Supplementary Materials (Supplementary Protocol 6). Individual lysates, each containing 10 µg of protein, were merged, and the obtained mixtures were subjected to in-solution trypsin digestion according to Supplementary Protocol 7, and subsequently to LC-MALDI MS/MS protein identification (8 µg of peptides per injection). The tryptophan fluorescence method by Wiśniewski and Gaugaz [37] was employed for protein/peptide quantitation. The hypothetical identity of the MSI components was established by the assignment of a component location on the mass/charge scale for the measured masses of tryptic peptides identified by LC-MS/MS allowing ±0.05% mass tolerance. Complete LC-MALDI-MS/MS data are available in the ProteomeXchange/PRIDE repository (dataset ID: PXD027878).

## 3. Results

**Figure 1:**
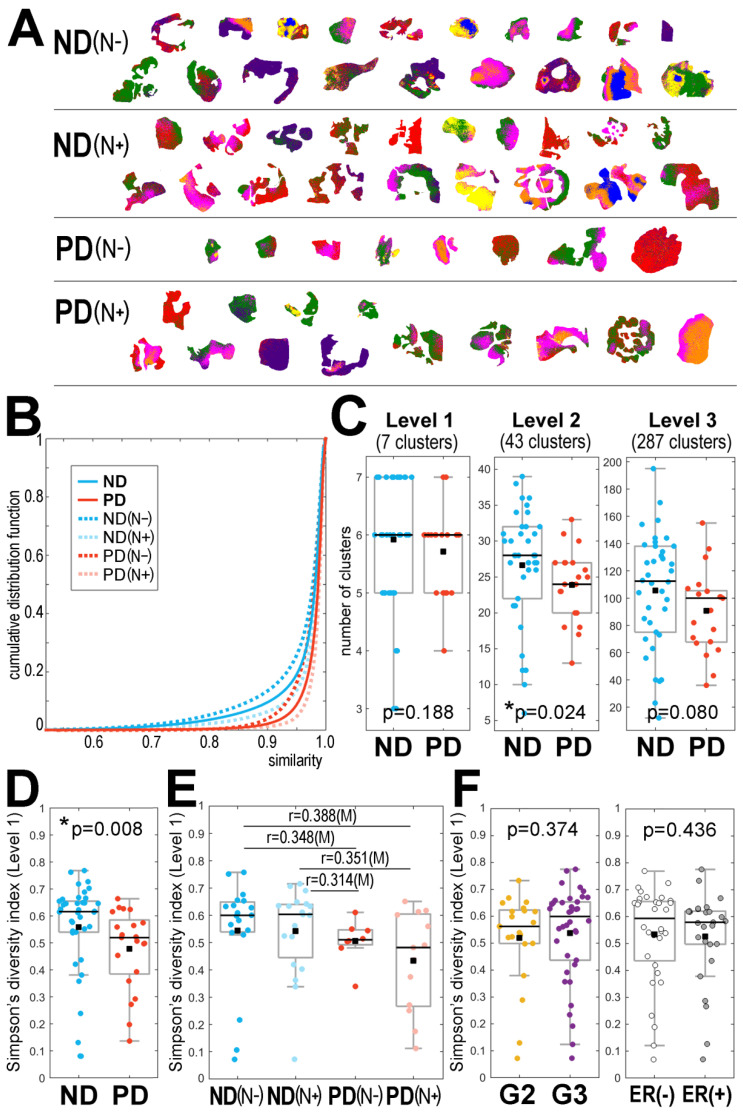
Heterogeneity of cancer ROI revealed by MALDI-MSI in breast cancer. Panel (**A**)—the distribution of 7 clusters (artificially color-coded) defined at the first level of unsupervised segmentation of cancer ROIs from 59 tissue specimens analyzed together; patient groups: ND—no evidence of disease, PD—progressive disease, cancer-free and cancer-containing lymph nodes marked with (N−) and (N+), respectively. Panel (**B**)—the cumulative distribution function of the spectra similarity index analyzed within each cancer ROI. Panel (**C**)—the number of clusters generated at the first three levels of image segmentation in samples from the ND and PD groups. Simpson’s diversity index computed for the first level of image segmentation in samples from the ND and PD groups (Panel (**D**)), samples from the patients’ subgroups with different lymph node status (Panel (**E**)), and samples of cancer with different pathological grades (G2 vs. G3) and expression of estrogen receptors (Panel (**F**)). Boxplots represent minimum, maximum, lower and upper quartile, and median. The *p*-value of differences between two groups (panels (**C**,**D**,**F**)) and [r] effect size for multiple pairwise comparisons of four groups is shown (panel (**E**); differences with at least a medium effect size are shown). * *p* < 0.05 is marked with asterisks.

This retrospective analysis included tissues resected surgically from 59 patients during their primary treatment due to a locally advanced HER2-positive invasive ductal carcinoma of the breast. Two major groups of patients were included: with no evidence of disease during a 60-month follow-up (ND; *n* = 38), and with distant metastases (progressive disease, PD; *n* = 21). In both groups, patients without synchronous lymph node metastases (N0) and patients with synchronous lymph-node metastases (N+) were distinguished (Table 1). Molecular maps of tryptic peptides were registered in sections of FFPE material by MALDI-MSI in a 600–3500 *m/z* range; 2527 spectral components were identified, which represented different peptide species with their isotope envelopes (the average mass spectrum is illustrated in Supplementary Figure S2). Cancer regions of interest (ROIs) were delineated by a pathologist in each specimen, and all spectra from these ROIs were used in further experiments (the size of ROIs ranged from 1768 to 14,327 spectra/pixels). Spectra from all cancer ROIs (377,310 spectra together) were clustered using the unsupervised procedure based on the iterative k-means algorithm of clustering with a data-driven optimization of cluster numbers [23,31]. The first three steps (levels) of such molecular image segmentation (which generated 7, 43, and 287 clusters, respectively) appeared the most informative; Figure 1A illustrates the distribution of seven clusters generated at the first level of the procedure. To assess the heterogeneity of the imaged tissues, we first analyzed the overall similarity of spectra within the cancer ROI of each patient. Figure 1B illustrates the cumulative distribution function of the similarity index calculated for cancer ROIs in each group of patients. We observed a very high level of similarity of spectra within each ROI (a median similarity index of about 0.98; Supplementary Table S1), which indicated a generally low heterogeneity of cancer tissue. Nevertheless, a slightly lower similarity of spectra (i.e., higher intra-ROI heterogeneity) was observed in the ND group compared to the PD group.

In the next step, we looked at the number and diversity of clusters generated by the unsupervised procedure that were present in the cancer ROI of each sample. In general, a positive correlation between the size of an ROI (i.e., the number of spectra) and the number of clusters was observed (Supplementary Figure S3A). Importantly, however, in all groups of patient sizes the ROIs were similar (Supplementary Figure S3B), and the comparison of the number of clusters between groups was credible. Figure 1C compares the number of clusters observed in the ND and PD samples after the first three steps of image segmentation (further levels of image segmentation are illustrated in Supplementary Figure S4A). We observed that at each segmentation level the number of clusters was generally higher in cancer ROIs from the ND group; this difference was statistically significant (*p* = 0.024) when the second level of image segmentation was analyzed.

To further compare the heterogeneity of cancer ROIs between the ND and PD groups, we took into account not only the number of clusters but also the size of each cluster. Simpson’s diversity index was applied, which estimates the probability that two pixels belong to different clusters; a higher index reflects a lower probability of co-clustering, which indicates a higher heterogeneity. Figure 1D compares Simpson’s diversity index calculated for the ND and PD samples after the first step (level) of image segmentation (further levels of image segmentation are illustrated in Supplementary Figure S4B). The diversity index was generally higher in cancer ROIs from the ND group (this difference was statistically significant at the first level of image segmentation, *p* = 0.008), which confirmed a higher level of intra-ROI heterogeneity in the ND group compared to the PD group. To further analyze this phenomenon, we compared subgroups of patients with different regional lymph node status; both the number of clusters and the diversity index were analyzed. Figure 1E compares the diversity index calculated for each patient subgroup at the first level of image segmentation (the relevant number of clusters at different segmentation levels is presented in Supplementary Figure S5A). It is noteworthy that the heterogeneity of cancers with and without simultaneous lymph node metastases, i.e., ND(N−) vs. ND(N+) and PD(N−) vs. PD(N+), was similar, and hence the difference between patients with a different outcome (i.e., ND vs. PD) remained the major observed effect. Finally, we also compared the heterogeneity of cancer ROIs in samples from patients with different cancer stages at the time of the initial diagnosis and treatment. Figure 1F shows that the diversity index was comparable in samples with a pathological grade G2 and G3. Moreover, differences in heterogeneity were not observed between specimens with different clinical cancer stages (Supplementary Figure S5B). Furthermore, the observed heterogeneity was not associated with the expression of estrogen receptors (Figure 1F). Therefore, in contrast to the long-term outcome, the initial stage and status of cancer were not associated with the heterogeneity revealed by MALDI-MSI (which in part reflected the rather high uniformity of cancer cases selected for the study). We also found that in the analyzed group neither the clinical cancer stage nor the pathological grade were associated with a long-term outcome. On the other hand, we observed a higher contribution of ER-positive cases in the PD group (*p* = 0.028). However, since the estrogen receptor status and the ITH level revealed by MSI did not correlate, both parameters seemed to be independently associated with a long-term outcome.

**Figure 2:**
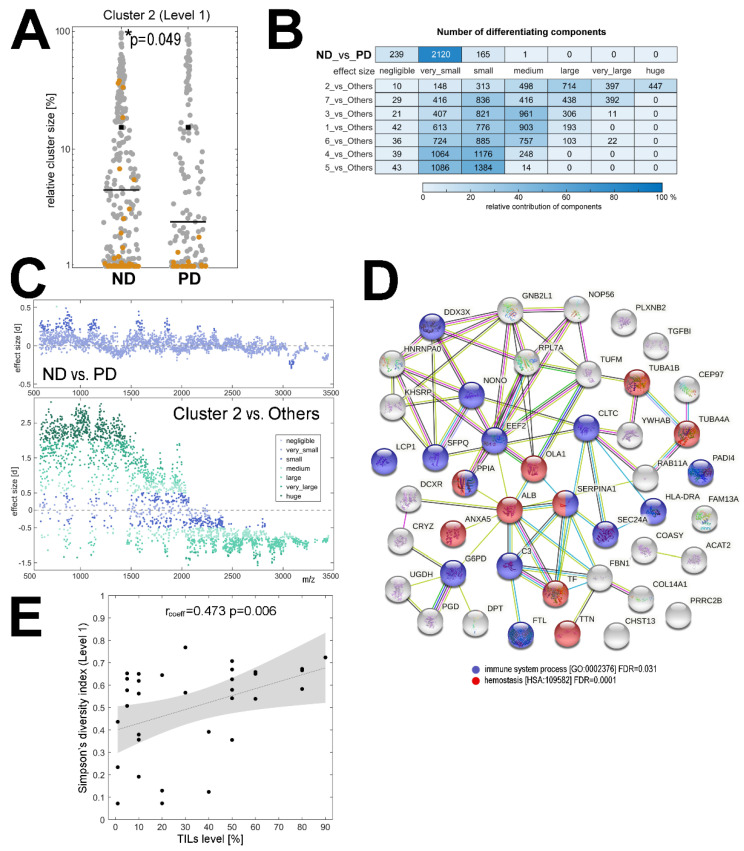
Components that differentiated samples of patients with a different outcome. Panel (**A**)—the relative contribution [%] of cluster #2 in the cancer ROI of samples from the ND and PD groups (cluster #2 is marked in brown, all other clusters are shown in grey). * *p* < 0.05 is marked with asterisks. Panel (**B**)—the number of spectral components with a different Cohen’s [d] effect size between the ND and PD groups as well as between a specific cluster (7 clusters from the first level of segmentation) and spectra from all other clusters together. Panel (**C**)—spectral components that had a different abundance between the ND and the PD groups (upper graph) and between cluster #2 and all other clusters (bottom graph); the graphs illustrate the molecular size (*m/z*) and the significance of the differences ([d] effect size; positive and negative values reflect the relative upregulation and downregulation). Panel (**D**)—the network of interactions between 45 proteins, the tryptic fragments of which were putatively identified as spectral components with different abundance between the ND and PD groups; interaction between proteins and over-represented biological functions associated with these proteins according to an analysis using the STRING toolbox [38]. Panel (**E**)—correlation between the level of tumor-infiltrating lymphocytes (TILs; shown as a percentage of the cancer area) and ITH revealed by MSI (Simpson’s diversity index at level 1 of image segmentation); the image shows the regression line with 95% confidence interval.

To further characterize the differences between the tumor samples collected before the treatment from patients who finally had a different outcome, we compared the structure of clusters distinguished by MALDI-MSI in the ND and PD groups. First, we analyzed the relative contribution of different clusters in cancer ROIs and found that cluster #2 (level 1 of the segmentation) had a significantly higher contribution in the cancer ROI of the ND samples than in that of the PD samples (Figure 2A). Then, we identified spectral components that showed a significantly different abundance between clusters (the Cohen’s effect size was used to determine the significance, which helps to overcome statistical problems potentially related to a very large number of analyzed spectra)—the number of differentiating components is illustrated in Figure 2B.

In general, we found numerous components whose abundance was markedly different between clusters (details in Supplementary Table S2). Cluster #2 (level 1 of the segmentation) had the most distinct characteristics: about 80% of all detected components showed significant differences in abundance (at least medium effect size) when compared to spectra from all other clusters together, which is illustrated in Figure 2C. On the other hand, if spectra from the overall cancer ROI were compared between the ND and PD samples, only a few components showed significant differences between groups (about 6% of the detected components had a small or medium effect size); Figure 2B,C. Therefore, to strengthen the information about components that indeed differentiated both groups of patients, we searched for components that simultaneously showed differences between cluster #2 and other clusters (at least medium effect size) and between the ND and PD groups (at least small effect size). This analysis revealed 120 spectral components (Supplementary Table S2) which corresponded to tryptic peptides that had a putatively different abundance in the tumor tissue between the ND and PD groups.

To extend this observation, a hypothetical identity of MSI components was established by attributing masses (*m/z* values) of spectral components (i.e., tryptic peptides) to the measured masses of peptides identified by the LC-MS/MS technique in lysates from cancerous tissue; the lists of the identified peptides and corresponding proteins are provided in Supplementary Tables S3 and S4, respectively. The matches between the identified peptides and the MSI components are presented in Supplementary Table S5; one should be aware, however, that this type of annotation is not unique, and more than one identified peptide could be matched to certain MSI components due to the relatively low resolution of MALDI-ToF MSI. Nevertheless, spectral components that could be considered as differentiating between cancer ROIs of the ND and the PD samples were putatively associated with 45 proteins (Supplementary Table S5), the potential functional network of which is illustrated in Figure 2D. It is noteworthy that among the overrepresented biological functions associated with this set of proteins there were immune-related processes and hemostasis (Figure 2D), which putatively reflected the presence of blood and immune cells in the component that differentiated tumors with different outcomes. This observation was followed with an additional analysis performed using information about the level of tumor-infiltrating lymphocytes (TILs), which was available for a subset of patients from the analyzed group (*n* = 33). A significant positive correlation between the level of TILs and the level of ITH assessed by MALDI-MSI was found (Figure 2E and Supplementary Figure S6A). Moreover, the level of TILs was higher in the ND group than in the PD group (medium effect size; Supplementary Figure S6B).

## 4. Discussion

The intra-tumor heterogeneity has been widely described in breast cancer [39,40]. This heterogeneity has been evidenced by next-generation sequencing at the level of tumor genome, which revealed clonal evolution and the co-existence of genetically distinct subclones of cancer cells [41,42,43,44]. Molecular ITH determined by genetic and epigenetic alterations is mirrored at the level of the phenotype of breast cancer cells, which is exemplified by the heterogeneity in the expression of the HER2 receptor caused by the heterogeneous distribution of *HER2* gene amplification [45,46]. However, with a marked exception of triple-negative breast cancers (TNBC) [44,47], the ITH observed in breast cancer is relatively low when compared to other cancers. For example, breast cancer was the least heterogenic in a set of nine cancer types when genetic ITH was estimated based on mathematical modeling of mutational datasets [11]. Interestingly, we found a high homogeneity of MALDI-MSI maps, which indicated a generally low heterogeneity in the analyzed group of HER2-positive tumors (the intra-tumor similarity of MSI spectra was higher than in previously analyzed thyroid cancers [48]). Though the majority of reports indicate that high genetic ITH is associated with a worse prognosis of breast cancer, it was also suggested that the prognostic value of tumor heterogeneity could be compromised by comorbidities present in elderly patients [49]. Nevertheless, according to the international guidelines for breast cancer treatment, the heterogeneity of the expression of diagnostic markers (e.g., HER2 or Ki67) should be taken into consideration when therapeutic decisions are made [50,51].

In this communication, we noted that a higher degree of intra-tumor heterogeneity was associated with a better prognosis in breast cancer. This observation was in marked contrast to the data presented by Morris et al. [11], who reported that a high degree of hypothetical genetic ITH modeled using the TCGA dataset was associated with an increased risk of reduced overall survival. However, a few important aspects of this particular observation should be noted. The large cohort of breast cancer patients included in that modeling-based study (*n* = 878) involved different histological and molecular subtypes of breast cancer (which was generally described as “breast invasive carcinoma”), and the majority of tumors (63%) had low heterogeneity (i.e., one dominant clonal population). The above-mentioned low-ITH cancers most probably comprise luminal A and B carcinomas, which constitute about 60–70% of all breast carcinomas that have a relatively favorable prognosis and are characterized by a rather low frequency of mutations [52]. On the other hand, high-ITH cancers might correspond to TNBC (with basal-like carcinomas as one of its subtypes) that have a much worse prognosis and are characterized by a significantly higher frequency of genetic aberrations and pathogenic single nucleotide mutations (*BRCA1/2*, *TP53*) than other types of breast carcinomas [40,44,53,54]. On the contrary, we analyzed only one breast cancer subtype (HER2-overexpressing) and selected only early breast cancer patients to whom radical local therapy was applied, followed by trastuzumab and chemotherapy/hormonotherapy in the adjuvant setting, which is linked with a relatively good overall prognosis [55,56].

Due to the analytical properties of MALDI-MSI, only the phenotypical heterogeneity of the tumor was studied, which reflected different phenomena if compared to the genetic heterogeneity of breast cancer addressed by the studies mentioned above. A tumor mass represents a complex network of genetically different (sub)clones of neoplastic cells (i.e., the source of genetic ITH), immune cells, vasculature cells, stromal cells, and an extracellular matrix, which together constitute the tumor microenvironment (TME) [5,57,58]. As a consequence, the morphological ITH of breast cancer results from different compositions of tumor stroma and/or different tumor/stroma ratios [59]. Therefore, clusters of these heterotypic components of TME could be revealed by MALDI-MSI. The immune system plays an important role in cancer progression, either by eliminating cancer cells or by stimulating tumor growth [60,61]. It is noteworthy that tumor infiltration by lymphocytes indicated an antitumor response of breast cancer, and the level of tumor-infiltrating lymphocytes (TILs) was associated with a better outcome in HER2-positive cancers [62,63]. Moreover, tumor infiltration by lymphocytes and other components of the immune system could have heterogeneous patterns, and the diffuse distribution of TILs could be a marker for better prognosis [64,65]. This particular aspect of intratumor heterogeneity was analyzed previously at a single-cell level using different multiplex antibody-based targeted approaches [66]. For example, it was shown that different spatial patterns of immune cell populations in the TME were associated with different outcomes in TNBC [67]. More recently, the heterogeneity of cancer- and immune-related proteins was compared in pre-treatment and on-treatment biopsy samples from HER2-positive cancers undergoing neoadjuvant HER2-targeting treatment, which revealed that the increased heterogeneity of immune-related proteins (e.g., pan-leukocyte marker CD45) after a single cycle of HER2-targeting agents was associated with a complete response [68]. Here we showed that a high level of TILs present in cancer was associated with a high degree of heterogeneity revealed by MALDI-MSI. Therefore, considering the supporting data from the literature, we hypothesized that the heterogeneity linked to the tumor infiltration by immune cells with anti-cancer activities could explain the observed correlation between a higher phenotypic ITH and a better outcome in a group of patients with HER2-positive breast cancers. Moreover, because a subset of proteins that putatively differentiated tumors with better and worse prognoses was associated with hemostasis, differential microvasculature could further contribute to the observed heterogeneity, providing another putative cause of different responses to treatment.

## 5. Conclusions

We unexpectedly noted that the higher degree of phenotypic heterogeneity revealed by mass spectrometry imaging in resected tumor tissue was associated with a favorable outcome in patients with locally advanced HER2-positive breast cancer treated with trastuzumab. Proteins involved in immune processes and hemostasis were putatively associated with the observed heterogeneity. Moreover, the observed heterogeneity correlated with the level of tumor-infiltrating lymphocytes. On the other hand, the detected heterogeneity was associated neither with tumor stage nor with lymph node status, pathological grade, or the expression of estrogen receptors. Therefore, we propose that a higher heterogeneity of tumors with a better prognosis could reflect the presence of heterotypic components in the tumor microenvironment, including infiltrating immune cells, that facilitated the response to treatment.

## Figures and Tables

**Table 1:** Clinical characteristics of patients included in the study.

Group	ND(N−)	ND(N+)	PD(N−)	PD(N+)
*n*	19	19	8	13
tumor size				
T1c	12	5	3	5
T2	7	14	5	8
lymph node status				
N0	19	-	8	-
N1	-	10	-	4
N2	-	3	-	7
N3	-	6	-	2
cancer stage				
IA	12	-	3	-
IIA	7	3	4	2
IIB	-	8	1	3
IIIA	-	2	-	6
IIIC	-	6	-	2
tumor grade				
G2	5	6	4	6
G3	14	13	4	7
hormone receptor				
ER(+)/PR(+)	4	3	4	8
ER(+)/PR(−)	2	4	0	2
ER(−)/PR(−)	13	12	4	3

ND—no evidence of disease; PD—progressive disease; (N0)—without synchronous lymph-node metastases; (N+)—with synchronous lymph-node metastases; ER—estrogen receptor, PG—progesterone receptor.

## Data Availability

The LC-MALDI-MS/MS-based proteomic data have been deposited in the ProteomeXchange Consortium via the PRIDE (https://www.ebi.ac.uk/pride, accessed on 12 August 2021) [69,70] partner repository with the dataset identifier PXD027878. The remaining data presented in this study are available on request from the corresponding author. The data are not publicly available due to privacy and ethical reasons.

## Supplementary Materials

The following are available online at https://www.mdpi.com/article/10.3390/cancers13174349/s1, Figure S1: Cancer Region of Interest (ROI) delineated in a tissue specimen, Figure S2: Average MALDI-ToF mass spectrum of the cancer ROI, Figure S3: Size of cancer ROI in tissue specimens analyzed by MALDI-MSI., Figure S4: Differences in heterogeneity of cancer ROI between the ND and PD groups of patients, Figure S5: Differences in heterogeneity of cancer ROI between patients’ groups, Figure S6: Tumor-Infiltrating Lymphocytes (TILs) in samples of breast cancer, Table S1: The intra-ROI similarity index for MSI spectra, Table S2: Significance of differences for the abundance of spectral components between clusters as well as between whole cancer ROIs of the ND and PD groups, Table S3: Peptides identified by LC-MALDI-MS/MS in breast cancers, Table S4: Proteins identified by LC-MALDI-MS/MS in breast cancers, Table S5: Hypothetical identity of MSI components based on their assignment to measured masses of tryptic peptides identified by LC-MS/MS, Supplementary Protocols.
