# Supplementary material for: Intra-Tumor Heterogeneity Revealed by Mass Spectrometry Imaging Is Associated with the Prognosis of Breast Cancer
Source: Cancers (Basel). 2021 Aug 27;13(17):4349. doi: 10.3390/cancers13174349 (PMC8431441; doi:10.3390/cancers13174349)
Supplement: Supplementary file 1 [file cancers-13-04349-s001.zip › Supplementary Materials/Supplementary Figures S1-S6.pdf]

Intra-tumor heterogeneity revealed by mass spectrometry imaging is associated with the prognosis of breast cancer

Marta Gawin *et al.*

Supplementary Figures

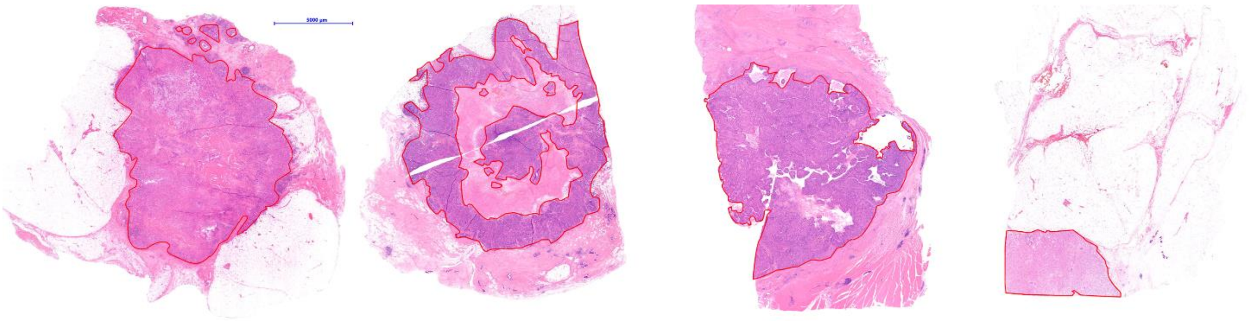

Figure S1. Cancer Region of Interest (ROI) delineated in a tissue specimen. Examples of tissue sections stained with hematoxylin and eosin (H&E) that were used to mark cancer ROI – tissue area within the red line; scale bar corresponds to 5,000 µm.

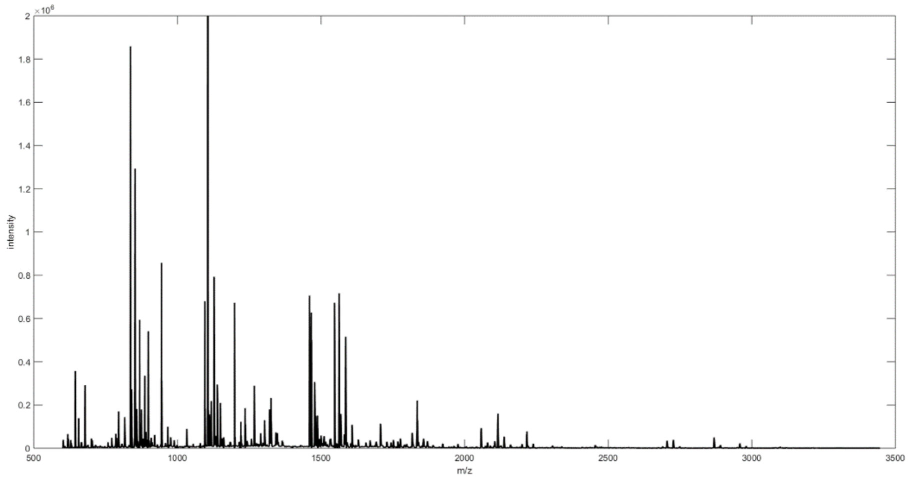

Figure S2. Average MALDI-ToF mass spectrum of the cancer ROI

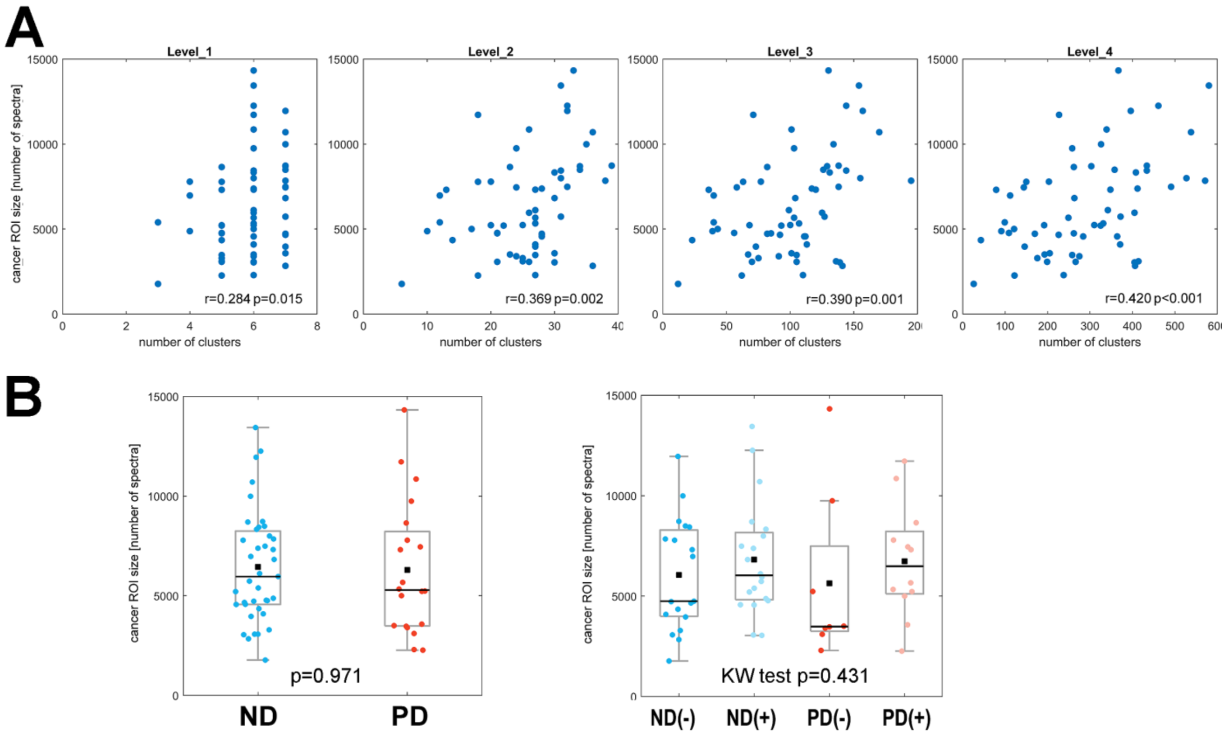

Figure S3. Size of cancer ROI in tissue specimens analyzed by MALDI-MSI. Panel A –correlation between the size of cancer ROI (number of spectra/image pixels) and the number of clusters generated at the first 4 levels of unsupervised image segmentation; the significance of correlation was assessed using a test based on Student's *t*-distribution. **Panel B** – the size of cancer ROIs compared between the ND and PD group (left) and between four subgroups of patients with different lymph node status (right). Boxplots represent minimum, maximum, median, lower and upper quartile; the significance of differences was assessed by the t-test and the Kruskal-Wallis test followed by the Conover’s post-hoc test for two and four groups, respectively.

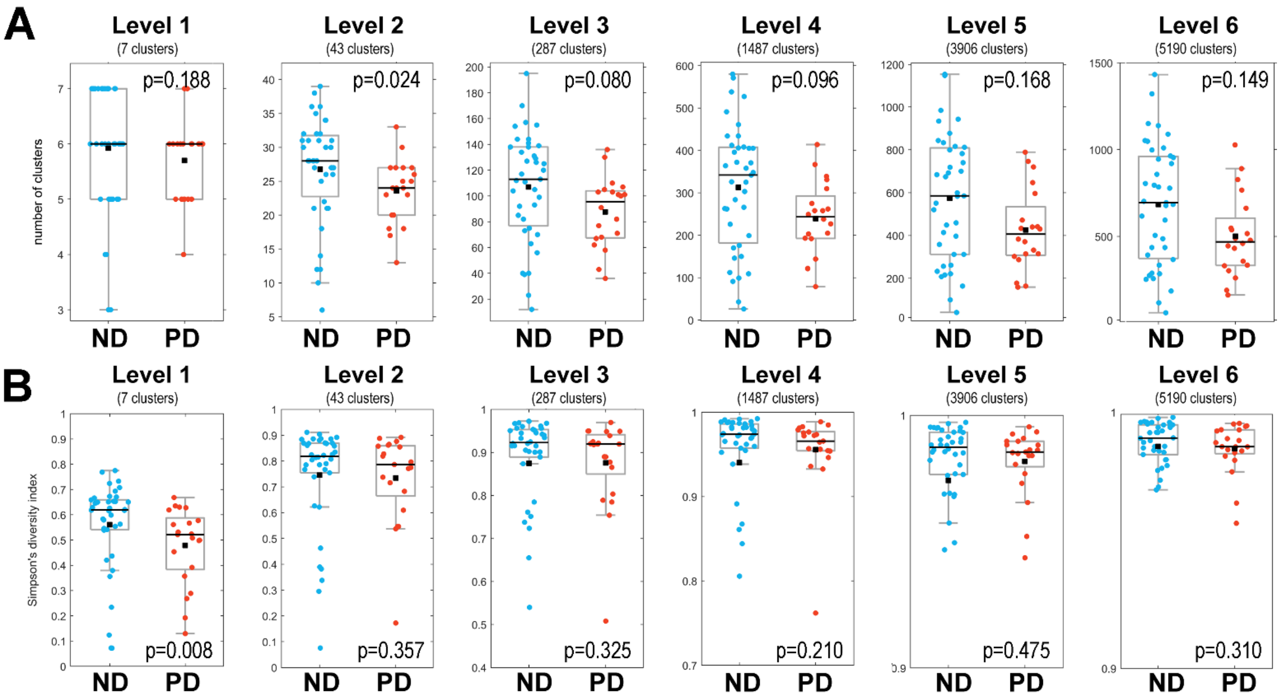

Figure S4. Differences in heterogeneity of cancer ROI between the ND and PD groups of patients. Panel A – the number of clusters generated at the first six levels of unsupervised image segmentation. **Panel B** – the Simpson's diversity index computed for the first six levels of image segmentation. Boxplots represent minimum, maximum, lower and upper quartile, and median. The significance of differences between the ND and PD groups was assessed by the Wilcoxon rank-sum test.

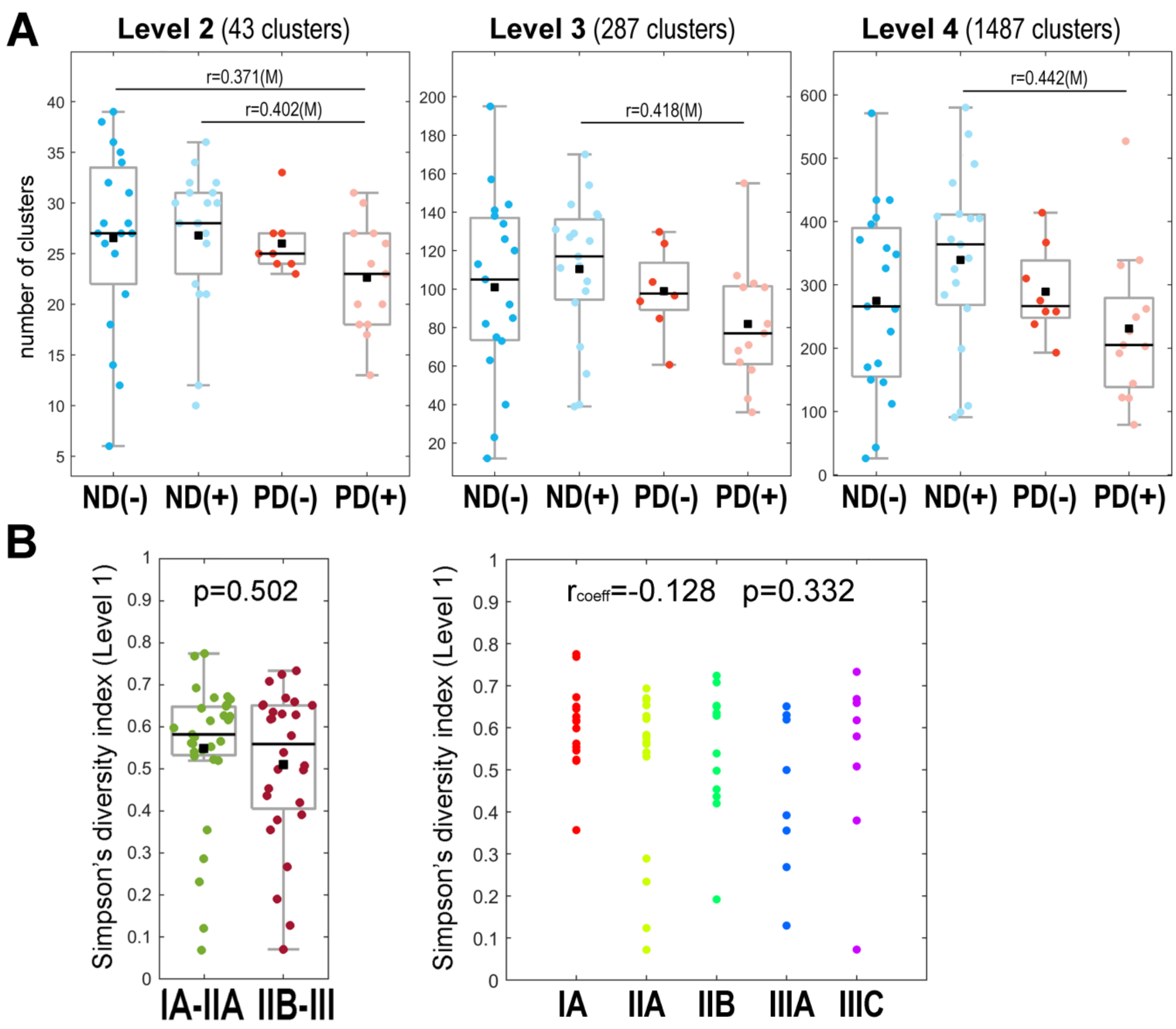

Figure S5. Differences in heterogeneity of cancer ROI between patients' groups. Panel A – the number of clusters generated at the second, third, and fourth level of unsupervised image segmentation in subgroups of patients with different regional lymph node status: (-) – without lymph node metastases, (+) – with synchronous lymph node metastases. Boxplots represent minimum, maximum, lower and upper quartile, and median. The significance of differences between the subgroups was assessed by the Kruskal-Wallis test followed by the post-hoc Conover's test for pairwise comparisons, which were complemented with the eta-squared effect size and [r] effect size, respectively. Marked is at least medium [r] effect size calculated for pairwise comparisons. **Panel B –** the Simpson's diversity index computed for the first level of image segmentation in groups of patients with different clinical cancer stages. The significance of differences between (IA-IIA) and (IIB-III) groups was assessed by the Wilcoxon rank-sum test (left). Hypothetical correlation between the Simpson's diversity index and clinical cancer stage was assessed by Spearman's rank correlation coefficient.

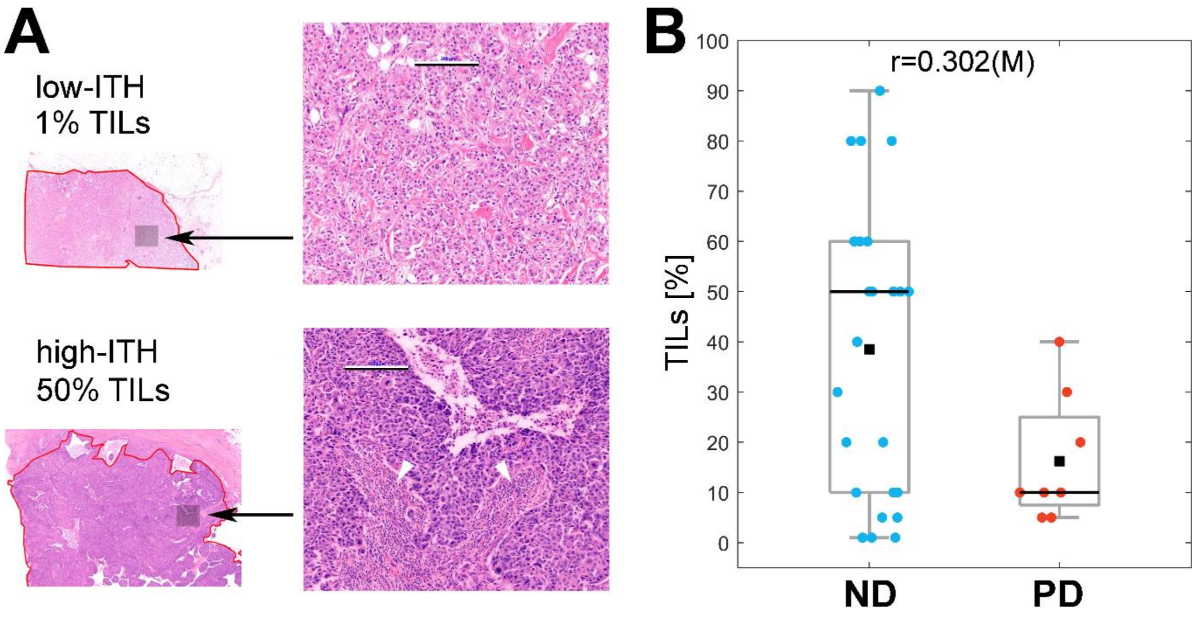

Figure S6. Tumor-Infiltrating Lymphocytes (TILs) in samples of breast cancer. Panel A – examples of tumor specimens with low and high levels of ITH and TILs (upper and bottom graphs, respectively). Scale bars in the inserts correspond to 200 µm; white arrowheads point at clusters of TILs. **Panel B –** differences in levels of TILs between the ND and PD groups. The level of TILs was estimated as a percentage of tumor area in each sample (*n*=33; ND=25, PD=8). The significance of differences between the groups was assessed by [r] effect size.
