# Supplementary material for: Intra-Tumor Heterogeneity Revealed by Mass Spectrometry Imaging Is Associated with the Prognosis of Breast Cancer
Source: Cancers (Basel). 2021 Aug 27;13(17):4349. doi: 10.3390/cancers13174349 (PMC8431441; doi:10.3390/cancers13174349)
Supplement: Supplementary file 1 [file cancers-13-04349-s001.zip › Supplementary Materials/Supplementary_protocols.pdf]

Gawin *et al.*: Intra-tumor heterogeneity revealed by mass spectrometry imaging is associated with the prognosis of breast cancer.

SUPPLEMENTARY PROTOCOLS

Supplementary Protocol 1: Paraffin removal procedure

Step	Washing solvent	Time of washing
1	100% xylene	5 min
2	100% xylene	5 min
3	99.8% ethanol	5 min
4	99.8% ethanol	5 min
5	96% ethanol	5 min
6	70% ethanol	5 min

Supplementary Protocol 2: Heat-induced antigen retrieval (HIAR) procedure

**Instrument**	Decloaking Chamber NxGen, Biocare Medical
**Retrieval solution**	DAKO REAL Target Retrieval Solution
**Program**	80°C, 2h
**Cooling**	on the bench: 20 min in the retrieval solution
**Washing**	MilliQ water, 2 x 1 min
**Drying**	on the bench: 20 min<br>in a vacuum desiccator: 40 min

Supplementary Protocol 3: SunCollect settings for trypsin and matrix deposition

Parameter	Trypsin deposition [1)]	Matrix deposition [2)]
Line distance [mm]	0.5	0.5
Z [mm]	35.0	20.0
Z offset [mm]	15.0	15.0
No. layers	4	8
Flowrate 1 [µL/min]	10	10
Flowrate 2 [µL/min]	10	20
Flowrate 3 [µL/min]	10	30
Flowrate 4 [µL/min]	10	40
Speed X [mm/min]	630 (= Low (9))	630 (= Low (9))
Speed Y [mm/min]	900 (= Med (1))	900 (= Med (1))
W [µg/mm^2] [3)]	0.0063	4.13

[1)] Sequencing grade modified trypsin (Promega): 0.05 µg/µL in 10 mM NH_4HCO_3, 10% ACN

[2)] HCCA (Bruker): 5 mg/mL in 60% ACN, 0.3% TFA

[3)] Amount of deposited trypsin/matrix

Supplementary Protocol 4: Spectra Processing Procedures.

The acquired MSI spectra were processed by applying a series of computational steps:

(i) The preprocessing module includes:

- resampling to common mass channels – mass channels unification across the MSI dataset using MatLab msresample() function from Bioinformatics Toolbox;
- baseline subtraction – the baseline was estimated within multiple shifted windows of adaptive width with quantile method to likely find the estimated baseline points; the window estimated points were regressed to a soft curve using spline interpolation [1];
- outlying spectra identification according to TIC value (as those with too high or too low TIC) with the use of Bruffaerts' criterion for skewed distributions [2];
- spectra alignment to reference spectrum based on Fast Fourier Transformation [3]; reference spectrum specified as the average spectrum of the MSI dataset with outlying spectra excluded;
- TIC normalization.

(ii) After preprocessing steps, the GMM procedure was applied for the average spectrum modeling and peak detection [4-6]. GMM for mass spectra processing enables precise representation of spectral peaks. Components of the GMM model of mass spectra are characterized by both positions and shapes (widths), while in most peak detection methods the information on shapes is missing requiring peak shift compensation and m/z intervals establishment. GMM spectra representation allows operating on exact m/z values.

(iii) A post-processing procedure was applied to reduce the data dimensionality by filtering out GMM components of high variance and low amplitude (components in the noise range). The thresholds for delineating the ranges of components of low amplitude and high variance were determined adaptively based on the data.

(iv) Following the GMM modeling, components representing the same peaks were identified and merged. Component abundance in individual spectra was estimated by pairwise convolution of the GMM model with preprocessed individual spectra.

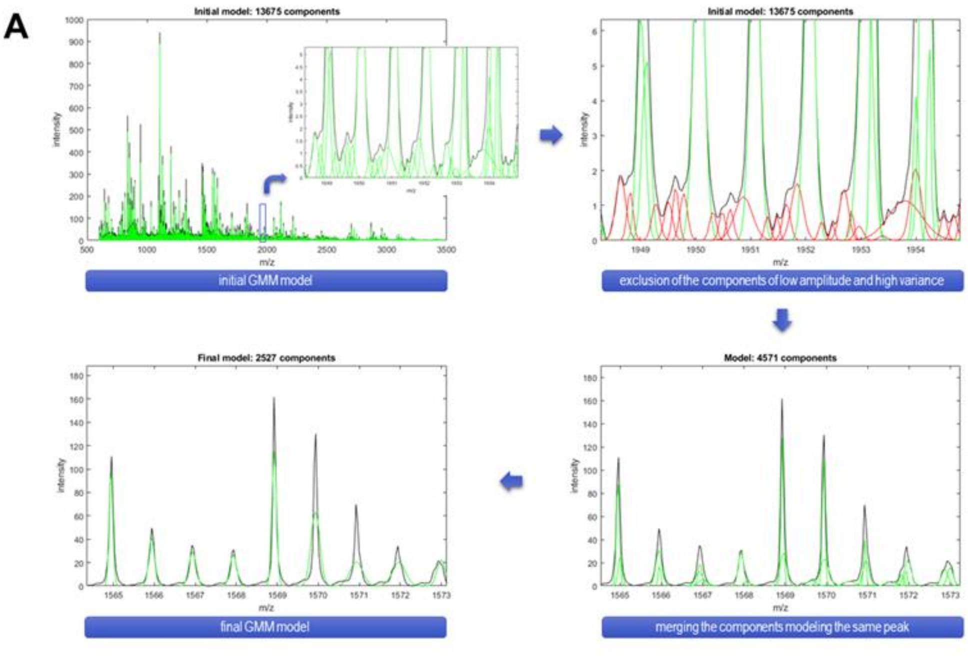

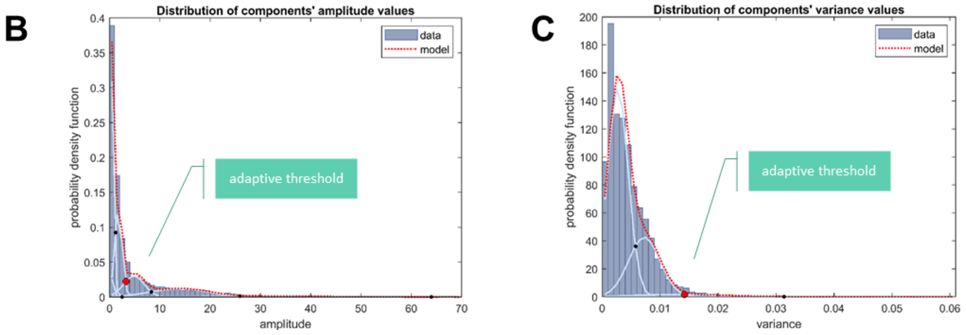

Figure SP1. The Gaussian mixture model (GMM) of a mass spectrum. Panel A – post-processing steps for initial GMM model; Average spectrum of the MSI dataset is plotted with black line and GMM components with a green line. GMM components excluded from the initial model during the post-processing procedure are red color-coded. Determination of adaptive thresholds to define the ranges of components of low amplitude (Panel B) and high variance (Panel C).

References

1. Bednarczyk, K.; Gawin, M.; Chekan, M.; Kurczyk, A.; Mrukwa, G.; Pietrowska, M.; Polanska, J.; Widlak, P. Discrimination of normal oral mucosa from oral cancer by mass spectrometry imaging of proteins and lipids. *J Mol Histol* **2019**, *50*, 1-10, doi:10.1007/s10735-018-9802-3.
2. Bruffaerts, C.; Verardi, V.; Vermandele, C. A generalized boxplot for skewed and heavy-tailed distributions. *Statistics & Probability Letters* **2014**, *95*, 110-117, doi:10.1016/j.spl.2014.08.016.
3. Wong, J.W.; Durante, C.; Cartwright, H.M. Application of fast Fourier transform cross-correlation for the alignment of large chromatographic and spectral datasets. *Anal Chem* **2005**, *77*, 5655-5661, doi:10.1021/ac050619p.
4. Pietrowska, M.; Marczak, L.; Polanska, J.; Behrendt, K.; Nowicka, E.; Walaszczyk, A.; Chmura, A.; Deja, R.; Stobiecki, M.; Polanski, A.; et al. Mass spectrometry-based serum proteome pattern analysis in molecular diagnostics of early stage breast cancer. *J Transl Med* **2009**, *7*, 60, doi:10.1186/1479-5876-7-60.
5. Polanski, A.; Marczyk, M.; Pietrowska, M.; Widlak, P.; Polanska, J. Signal Partitioning Algorithm for Highly Efficient Gaussian Mixture Modeling in Mass Spectrometry. *PLoS One* **2015**, *10*, e0134256, doi:10.1371/journal.pone.0134256.
6. Polanski, A.; Marczyk, M.; Pietrowska, M.; Widlak, P.; Polanska, J. Initializing the EM Algorithm for Univariate Gaussian, Multi-Component, Heteroscedastic Mixture Models by Dynamic Programming Partitions. *International Journal of Computational Methods* **2018**, *15*, 1850012, doi:10.1142/S0219876218500123.

Supplementary Protocol 5: Image Segmentation Procedures

The deglomerative divisive k-means (DivIK) algorithm with region-driven feature selection was applied for unsupervised molecular image segmentation [7,8]. DivIK is a framework developed for automated segmentation of multidimensional data. It splits data from the most general set to the most specific subregions and before each split it selects features with the greatest differentiating potential in a data-driven manner, to make the segmentation space adjusted to the specifics of a current subregion. The core of DivIK is based on k-means clustering methods. After the first sample split, the k-means algorithm is applied independently to each subregion obtained in the previous split and performs spectra division into an optimal number of clusters (Figure SP2). The optimal number of clusters is determined based on a quality measure, which compares the separation of clusters for a set of multiple segmentation maps with a different number of clusters. K-means clustering was performed for 2 to 10 clusters for every subregion and Dunn's index was calculated for the selection of the optimal number of clusters for which the subregion was finally split. Dunn's index computes a ratio of the minimal distance between clusters to the maximal distance inside a cluster. Spectra from all 59 cancer ROIs were analyzed together. The algorithm's stop condition was adjusted to complete segmentation when clusters consisting of less than or equal to 100 spectra were created.

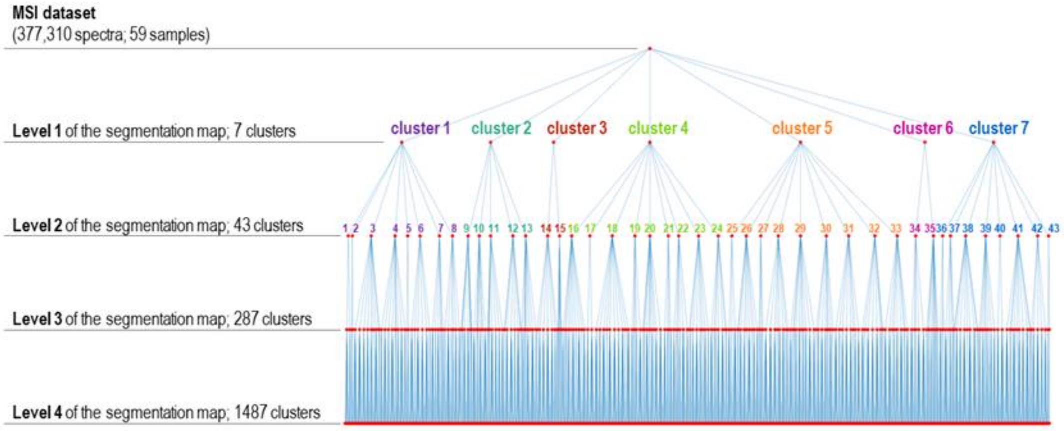

Figure SP2. The first four steps of the molecular image segmentation procedure that was used to generate clusters in all 59 samples.

References

7. Mrukwa, G.; Drazek, G.; Pietrowska, M.; Widlak, P.; Polanska, J. A Novel Divisive iK-Means Algorithm with Region-Driven Feature Selection as a Tool for Automated Detection of Tumour Heterogeneity in MALDI IMS Experiments. In Proceedings of the Bioinformatics and Biomedical Engineering, Cham, 2016; pp. 113-124.
8. Widlak, P.; Mrukwa, G.; Kalinowska, M.; Pietrowska, M.; Chekan, M.; Wierzgon, J.; Gawin, M.; Drazek, G.; Polanska, J. Detection of molecular signatures of oral squamous cell carcinoma and normal epithelium - application of a novel methodology for unsupervised segmentation of imaging mass spectrometry data. *Proteomics* **2016**, *16*, 1613-1621, doi:10.1002/pmic.201500458.

Supplementary Protocol 6: Preparation of tissue lysates for LC-MALDI-MS/MS protein identification.

1. FFPE tissue material collected in each Eppendorf tube was dewaxed with 1 mL of n-heptane (1h incubation at room temperature), then 50 µL of methanol were added; the tubes were subsequently centrifuged (16’000 RCF, 5 min) and supernatants were discarded.
2. Proteins were released from the tissue with 400 µL of tissue lysis buffer (0.1M Tris-HCl pH 8.0, 0.1M DTT, 4% SDS) upon heating at 99°C for 20 min, followed by incubation at 80°C for another 2h with constant mixing (750 rpm). The samples were then cooled down (4°C, 1 min), centrifuged at 16’000 RCF (45 min, 4°C) and supernatants were transferred to new tubes.
3. Proteins were subsequently precipitated from the supernatants with 10% (m/v) trichloroacetic acid (TCA) in acetone (freezing at -20°C, 18h). Protein pellets obtained after centrifugation (16’000 RCF, 30 min, 4°C) were washed twice with 500 µL of pure acetone (each washing followed by centrifugation at 16’000 RCF, 30 min, 4°C) and residues of acetone were evaporated at room temperature.
4. Pellets were then dissolved in 0.1% RapiGest SF (Waters) in 50 mM NH_4HCO_3 followed by boiling at 99°C for 10 min. Thus obtained samples were subjected to protein assay with the use of the tryptophan fluorescence method.

Supplementary Protocol 7: Preparation of protein digests for LC-MALDI-MS/MS protein identification

1. Proteins present in the lysate mixtures were subjected to disulfide bond cleavage with dithiothreitol (final concentration of DTT: 5 mM) with heating at 60°C for 30 min.
2. Alkylation was performed with iodoacetamide (final concentration of IAA: 15 mM) for 30 min in darkness (room temperature).
3. Sequencing Grade Modified Trypsin (Promega) was employed for proteolytic digestion with the enzyme to protein ratio of 1:50 (m/m). Incubation was performed for 18 h at 37°C.
4. Thus obtained digests were subsequently acidified with trifluoroacetic acid (final TFA concentration: 1%, v/v) and incubated at 37°C for 45 min. Then, the samples were centrifuged at 14’500 RCF for 20 min at room temperature and supernatants were transferred to new tubes.
5. Next, tryptic peptides were purified with the use of TT2C18 TopTips (Glygen Corp.) containing 10 mg of C18 bed with a particle diameter of 12 µm. SpinColumn-in-a-Tip mode was employed according to the manufacturer’s instructions with centrifugation at 2’150 RCF during column pre-conditioning and bed washing, and at 350 RCF during sample loading and elution.
6. Eluates were evaporated to dryness with the use of a vacuum centrifuge, reconstituted in 20 µL of LC-MS grade water, and subjected to peptide assay with the use of tryptophan fluorescence method.
7. Before LC-MALDI analysis samples were acidified with 2 µL of 1 % TFA (v/v).
